# Erratum: Causes and Consequences of Bacteriophage Diversification via Genetic Exchanges across Lifestyles and Bacterial Taxa

**DOI:** 10.1093/molbev/msab082

**Published:** 2021-04-12

**Authors:** Jorge A Moura de Sousa, Eugen Pfeifer, Marie Touchon, Eduardo P C Rocha

*Mol. Biol. Evol.* doi:10.1093/molbev/msab044

Supplementary data files unrelated to this article were published alongside this paper. The correct supplementary data files have now replaced the incorrect files online.

